# What coloration brings: Implications of background adaptation to oxidative stress in anurans

**DOI:** 10.1186/s12983-023-00486-z

**Published:** 2023-01-31

**Authors:** Tijana B. Radovanović, Tamara G. Petrović, Branka R. Gavrilović, Svetlana G. Despotović, Jelena P. Gavrić, Ana Kijanović, Marko Mirč, Nataša Tomašević Kolarov, Tanja Vukov, Marko D. Prokić

**Affiliations:** 1 Department of Physiology, Institute for Biological Research “Siniša Stanković”, National Institute of the Republic of Serbia, University of Belgrade, Bulevar despota Stefana 142, Belgrade, 11060 Serbia; 2 Department of Evolutionary Biology, Institute for Biological Research “Siniša Stanković”, National Institute of the Republic of Serbia, University of Belgrade, Bulevar despota Stefana 142, Belgrade, 11060 Serbia

**Keywords:** Amphibian larvae, Background color change, Adaptive plasticity, Physiological cost, Oxidative damage, Antioxidant system

## Abstract

**Background:**

Urban development results in habitat destruction, affecting populations of amphibians, the most fragile group of vertebrates. With changes in the environment, these animals become more exposed to light and predators. To enhance their chances of survival, they display plasticity of body coloration. Aside from adaptive benefits, animals exhibiting background matching meet the energetic costs and restrictions of changing body tones. To study the physiological consequences of *Hyla arborea* tadpole adaptation to background color, we followed oxidative stress parameters after rearing larvae on a constant background (black/white) and after changing the background color.

**Results:**

Larvae cultivated for 20 days on constant substrate color exhibited differences in body coloration but without differences in lipid peroxidation (LPO) concentration between dark and pale individuals, suggesting that coloration investment during this period did not induce higher oxidative damage in darker tadpoles. Prolonged exposure of larvae (37 days) to a dark habitat increased antioxidative system defense and LPO concentrations, compared to animals reared permanently in the white surroundings. The positive correlation of oxidative damage with color intensity of individuals points to the physiological consequences of higher investment in the number of pigment cells necessary for dark pigmentation. In individuals faced with non-matching background and change in body coloration, defense system declined and LPO occurred relative to individuals cultivated in white habitat.

**Conclusion:**

Here, we have pointed to consequences related to background matching and stress that amphibians experienced during chromatic adaptations. Background color change causes a complex physiological response affecting the antioxidative defense parameters. This investigation elucidates the accompanying cost of amphibiansʼ adjustment to an altered environment.

## Background

Natural habitats are greatly altered by anthropogenic activities, which lead to increased pollution and climate change. Urbanization and industrialization result in accelerated habitat loss. The outcome can be vegetation fragmentation or the development of gaps in a formerly contiguous habitat. Changes in forest cover near aquatic areas could be quite important for local amphibians. This process of vegetation loss increases the risk of animals being detected or recognized by natural predators [1]. Therefore, animals are faced with new challenges that affect their survival. The ability of some animal species to modify the phenotype, because of changes in environmental conditions, is considered as adaptive plasticity [2–4]. This capacity enables some organisms to change their natural body coloration and pattern (metachrosis) as an adaptation to a non-matching background. With adaptive plasticity organisms can improve performance when faced with pressures such as climate change, UV radiation, chemical pollutants and predators [5].

Depending on the species, alterations in body color can develop in different time ranges, from a couple of seconds to over a month [6, 7]. Physiological color change occurs in a shorter time interval of a few seconds, minutes or hours and is based on the dispersion and clustering of pigment inside cells. Changes that happen over days and weeks, due to the production and reorganization of pigment cells in the dermis, are frequently called morphological or quantitative [7, 8] and are usually linked to expected, long-term variations in the environment [6]. According to [9, 10] prolonged background adaptation (lasting for several days) leads to prolonged physiological color change, which precedes the morphological changes of color.

For amphibians, cryptic coloration can be the initial defense line and a form of antipredator adaptation [11]. By adapting to the color of the substrate and their environment, amphibians become less visible to predators, thereby increasing their fitness and survival chances. The proportion of this adaptive plasticity during ontogenesis is age-related as adult individuals exhibit lower plasticity than juveniles [6, 12]. The process of color change in amphibians is enabled by three types of dermal chromatophores (xanthophores, iridophores, and melanophores) [13]. When individuals are exposed to a different background color, a reaction-relocation of pigment organelles (melanosomes) in the dermal melanophores occurs. On a light background, pigment aggregation in melanophores results in skin lightening, while a dark background leads to pigment dispersion and darkening of the dorsal skin. Chromatophores modify their dimensions and density when chromatic adjustment lasts a week or more [14]. This color change is a reversible process in amphibians.

Intracellular transport of pigments is mediated by different hormones. Alpha-melanocyte stimulating hormone (α-MSH) induces the dispersion of melanin in melanophores that, then cover other color cells. In contrast, melanin-concentrating hormone (MCH) leads to the aggregation of pigment that causes lightening of the skin tone [15]. Besides these two primary hormones, pigment modifications in some amphibian species can also be affected by adrenaline, noradrenaline, progesterone and testosterone [16].

Chromatic adaptation, despite its benefits, also has an inevitable impact on other body processes and energy budgets and is linked to costs and restrictions. Color change through pigment reorganization or *de novo* synthesis involves complex neuroendocrine control of the chromatophores and may be followed by high energetic costs and metabolic activities [15]. These metabolic expenditures are the result of the many physiological reactions required for this adaptive response [17]. In color-changing animals, energy constraints are regulated by incidence and the category of change itself, thus, moderate change, based on diet, is less costly than rapid and continuous variations [12]. Amphibians select habitats that match their own coloration to maximize camouflage. The strong preference for certain backgrounds indicates that individuals try to avoid unnecessary color change, consequently reducing the involved energy costs [12, 15].

Even though changes in body color induced by changes in the environment (background matching) are associated with an increased metabolic rate, oxidative phosphorylation and, consequently, increased production of reactive oxygen species (ROS) [5], how a change in body color affects the physiology and antioxidant defense in anurans has not been examined. ROS disrupt the structure and function of macromolecules, cause tissue injury and have a detrimental influence on overall fitness. Therefore, the antioxidant protective apparatus must limit and neutralize their prooxidant activity and convert ROS into less harmful molecules [18].

**Fig. 1:**
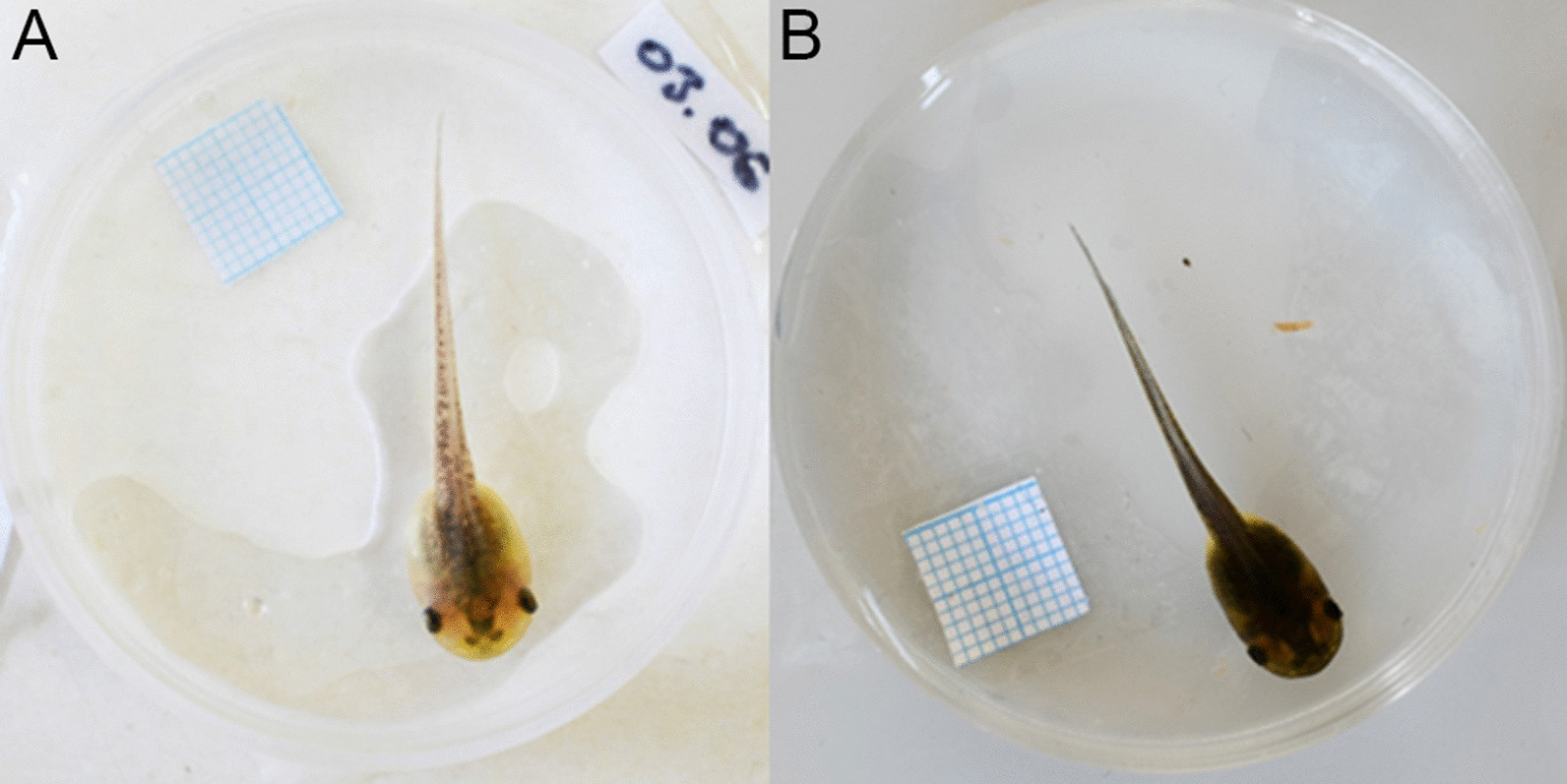
Tadpoles of *Hyla arborea* developed on a white (**A**) and black (**B**) background

The model organism for our experiment was the *Hyla arborea* tadpole (Fig. 1). Throughout the aquatic stage of life, amphibians encounter a variety of different invertebrate and vertebrate predators [19, 20]. Amphibian larvae frequently alter their morphology in response to predators, starting with the size, shape and color of their tails. For *H. arborea* tadpoles, dragonfly nymphs are a serious threat to their survival and can induce a plastic phenotypic response. Tadpoles developed deeper tails with black pigmented patches and changed their swimming behavior when predatory dragonfly larvae were present [21–23]. Energy investment in color change due to predatory risk represents an additional challenge for larvae which are already confronted with serious physiological, morphological and behavioral transformations essential for the crossover to a terrestrial habitat.

This study aimed to examine whether there are significant physiological consequences of changing coloration. We examined the evidence for the possible oxidative cost of adaptation of *H. arborea* tadpoles to background color. We assumed that: (i) tadpoles will adjust their body pigmentation in response to a change in background color; (ii) greater investment in pigmentation and its maintenance in darker larvae will result in an oxidative cost manifested through changes in oxidative status and induction of lipid peroxidation (LPO) due to an increase in ROS generated by a higher metabolic rate; (iii) exposure of individuals to a background color that is opposite to their body coloration (inadequate matching) will induce processes of color change, stress response and result in oxidative damage. The oxidative cost was assessed through changes in antioxidative system parameters and oxidative damage levels.

## Materials and methods

### Experimental design

Two hundred *Hyla arborea* eggs were collected from Reva pond, located in the vicinity of Belgrade, Serbia, (44° 50′53.8′′ N, 20° 32′07.7′′ E (44.848272, 20.535476)) during April 2021. The sample was transported in 1 L transparent containers (15.5·14.5·6.5 cm) and reared at the Department of Evolutionary Biology, Institute for Biological Research “Siniša Stanković”, University of Belgrade. The experiment started when tadpoles reached Gosner stage 25 (GS 25) [24]. In total, 120 specimens were randomly chosen for the experiment. Tadpoles were individually placed in plastic 0.5 L containers filled with dechlorinated tap water and reared at a constant room temperature (20 °C) under a natural day and night cycle. Water in the containers was changed every four days, and the tadpoles were fed *ad libitum* every other day with the same amount of pulverized commercial fish food tablets (Tetra TabiMin^®^, Tetra GmbH, Melle, Germany). The survival and general health of the tadpoles were visually inspected. The well-being of the animals was checked daily.

All surplus specimens (30 eggs) were returned to their natural habitat, 50 eggs never developed. Sixty containers had a black paper sleeve and were placed on a black surface (B—black background coloration treatment) and 60 containers had a white paper sleeve and were placed on a white surface (W – white background coloration treatment).

**Fig. 2:**
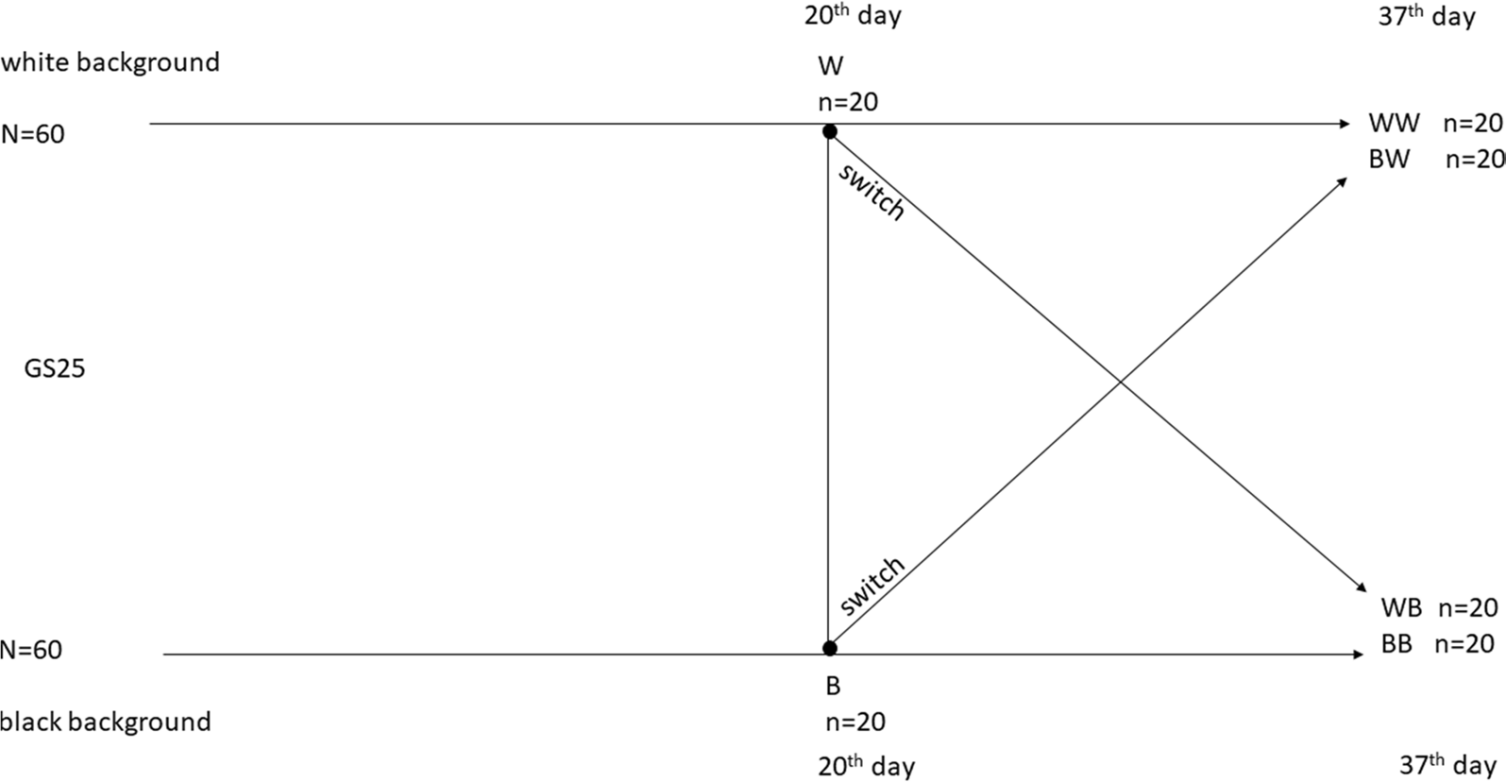
Schematic presentation of the experimental design

On the 20th day of the experiment 20 animals from the black surface (B treatment), and 20 animals from the white surface (W treatment) were randomly chosen and euthanized for analysis of their biochemical parameters of oxidative stress. Specimens were euthanized by submerging in liquid nitrogen. From the remaining 80 tadpoles, 20 animals from the B treatment were switched to the white containers (BW treatment) and the same number of animals from the W treatment were switched to the black containers (WB treatment). The remaining 40 animals resumed development under the original conditions (now BB and WW treatments, 20 animals each). Thereby four treatment groups were established. At the moment of the switch, the tadpoles were at GS 30 (Fig. 2).

On the 37th day of the experiment (17 days after the switch), all individuals from the treatment groups were euthanized in the same way as mentioned before, to analyze the parameters of oxidative stress caused by prolonged background adaptation. At this point, the tadpoles were at GS 37.

On the 20th and 37th days of the experiment, tadpoles were photographed to obtain length measurements. All photographs contained a ruler to standardize length measurements. Using the tpsDig program [25] we placed markers on the tip of the snout and the tip of the tail of tadpoles. Two markers 10 mm apart were placed on the ruler. Then, using tmorphgen from the Integrated Morphometrics Program (IMP) package [26] we calculated the lengths of the tadpoles. Additionally, on the same days, high-resolution images of the dorsal side of the tadpoles were taken for analysis of the pigmentation. All images were created using a DSLR Nikon D7500 camera, mounted perpendicularly to the animals and at a fixed height.

Pigmentation was described as the percentage of dark pixels on a tadpole’s body surface, calculated from photographs in Adobe Photoshop CC 2015 (Adobe System Inc., 2015). Pixels were described as light or dark based on their mean RGB value. First, we defined the RGB threshold values for each group and date, as the mean RGB value calculated from a subsample of specimens from each group and for each date. Pixels with lower RGB values were described as light and those with higher values as dark. Second, from the Adobe Photoshop histogram, we read the percentages of light and dark pixels for every tadpole.

### Tissue processing

The total body of the tadpoles used in biochemical analyses was minced and mixed into one homogenous mass; 0.2 g of the tissue was separated for the determination of thiobarbituric acid-reactive substance (TBARS) concentration, and the remaining homogenous tissue mass was used for antioxidative system (AOS) parameter assessment. The TBARS method [27] included tissue homogenization and sonication in 10 volumes of ice-cold Tris-HCl buffer, pH 7.4. The obtained sonicates were centrifuged for 10 min at 10,000 × g at 4 °C in 40% trichloroacetic acid (TCA). The resulting supernatant was used LPO determination at 532 nm and was presented in nmol TBARS/mg tissue.

The remaining homogenous tissue mass was used for the analysis of antioxidant parameters. The tissue was homogenized at pH 7.4 in 25 mM sucrose buffer [28] with 10 mM Tris-HCl and 5 mM EDTA [29] using an Ultra-Turrax homogenizer (T-18, IKA-Werk, Germany). Subsequently, using a Sonopuls ultrasonic homogenizer (HD 2070, Bandelin Electronic, Germany) the homogenates were sonicated at 20 kHz for 30 s. A part of the sonicate was used for measurement of total glutathione (GSH) concentration, and the remaining sonicate was centrifuged at 100,000×g for 90 min (4 °C) [30]. The obtained supernatant was used for the estimation of additional biochemical parameters.

### Biochemical analyses

The Lowry method was used for the determination of the total protein concentration at 500 nm [31]. The activity of superoxide dismutase (SOD) was evaluated at 480 nm, by monitoring the autoxidation of adrenaline to adrenochrome [32]. The Claiborne method [33] that was used for catalase (CAT) activity determination is based on the quantification of hydrogen peroxide decomposition and was carried out at 240 nm. The assays for glutathione peroxidase (GSH-Px) and glutathione reductase (GR) activity were performed according to the methods of [34] and [35], respectively. The [36] procedure was used for the evaluation of glutathione-S-transferase (GST) activity by measuring the interaction of 1-chloro-2,4-dinitrobenzene (CDNB) with the sulfhydryl group of GSH. The activities of GSH-Px, GR, and GST were monitored at 340 nm. The activities of the enzymes were interpreted in U/mg protein.

The Griffith method [37] was applied for the analysis of total GSH concentration, based on the oxidation of GSH by 5,50-dithiobis-(2- nitrobenzoic acid (DTNB) and NADPH reduction. Total GSH concentration was measured at 412 nm and expressed as nmol/g tissue.

All investigated parameters were rated at 25 °C using an UV-VIS spectrophotometer (UV-1800, Shimadzu, Japan).

## Statistical analysis

The Grubbs test was applied to check and exclude outlier data (we did not detect any outlier). The data were verified with the Kolmogorov-Smirnov one-sample test and all investigated parameters had a normal distribution. To test total snout-vent length (SVL), color intensity and differences in oxidative stress components between the studied groups of tadpoles (between the B and W group on the 20th day, and among WW, WB, BW and BB groups on the 37th day), one-way analysis of variance (ANOVA) was implemented with the background color as an independent variable. For parameters that showed significant differences (on the 37th day), we applied the post hoc Fisher least significant difference (LSD) test to determine further differences. The significant level was determined at p < 0.05. Linear regression was used to observe the relationship between the intensity of pigmentation/coloration and oxidative stress damage (LPO) in individuals exposed constantly on a white and black background. We used discriminant analysis (DA) to identify the differences among the examined tadpole groups (WW, WB, BW and BB) on the 37th day based on all measured oxidative stress components. In all figures and tables, the obtained data are presented as the mean ± standard error (SE). All results were calculated with STATISTICA 8.0. Software, with exception of DA which was performed in XLSTAT, Ver. 2014.5.03.

## Results

The measured values for total snout-vent length (SVL) and color intensity (% of dark pixels) of *Hyla arborea* tadpoles in the experimental groups are given in Table 1. One-way ANOVA revealed that there were no significant differences between SVL values in W and B groups on the 20th day of the experiment (p = 0.6455). However, body pigmentation differed significantly between the two groups (p < 0.0001). For prolonged background adaptation, after 37 days of the experiment the Fisher LSD test revealed that individuals from the BB group were statistically longer than WB individuals (p = 0.0199). All groups displayed a statistically significant difference in pigmentation among themselves, except groups BB and WB (WW vs. WB–p < 0.0001; WW vs. BW– p = 0.0007; WW vs. BB– p < 0.0001; WB vs. BW– p < 0.0001; BB vs. BW–p < 0.0001).

**Table 1:** The total snout-vent length (SVL) and color intensity (% of dark pixels) of *Hyla arborea* tadpoles in experimental groups (W – white, B – black, WW – white-white, WB – white-black, BW – black-white and BB – black-black)

	Groups	Mean ± SE	W versus B	WW versus WB	WW versus BW	WW versus BB	WB versus BW	BB versus BW	BB versus WB
SVL (mm)	W B	32.07 ± 0.98 32.58 ± 0.51	p = 0.6455	p = 0.2059	p = 0.9646	p = 0.2870	p = 0.2022	p = 0.3220	**p = 0.0199**
	WW WB BW BB	46.96 ± 0.59 45.61 ± 0.75 47.01 ± 1.06 48.07 ± 0.59							
Color (dark pixels %)	W B	57.84 ± 2.74 92.29 ± 1.30	**p < 0.0001**	**p < 0.0001**	**p = 0.0007**	**p < 0.0001**	**p < 0.0001**	**p < 0.0001**	p = 0.7011
	WW WB BW BB	70.39 ± 2.59 91.41 ± 1.27 59.88 ± 2.19 90.41 ± 1.52							

The data are expressed as the mean ± SE. Data in bold indicate statistical differences (p < 0.05) based on one-way ANOVA with the Fisher LSD post hoc test

**Fig. 3:**
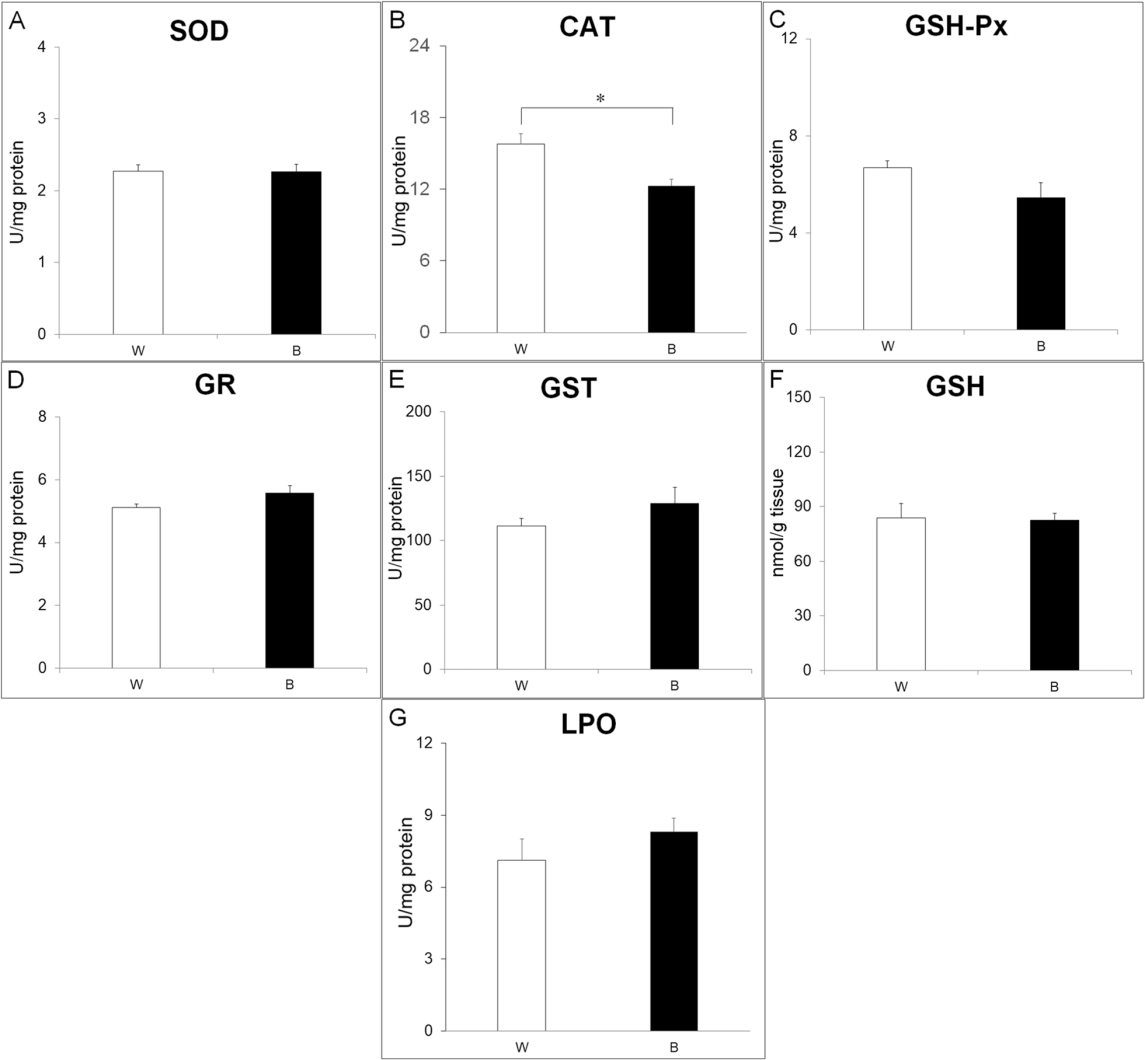
Antioxidant system (**A**—superoxide dismutase (SOD); **B—**catalase (CAT); **C—**glutathione peroxidase (GSH-Px); **D—**glutathione reductase (GR); **E—**glutathione-S-transferase (GST); **F—**glutathione (GSH)) and oxidative stress (**G**—lipid peroxidation (LPO)) parameters in two experimental groups (W—white, B—black) of *Hyla arborea* tadpoles. *indicates significant differences between groups (one-way ANOVA, p < 0.05)

The results for one-way ANOVA are given in Table 2. Only the activity of CAT was significantly reduced in the group of tadpoles that were constantly exposed to the black background (B) with respect to the W group (p = 0.0032). The other examined biochemical parameters did not differ between these two groups (Table 2; Fig. 3). One-way ANOVA showed significant differences for all oxidative stress parameters among WW, WB, BW and BB groups.

**Table 2:** Results of one-way ANOVA of the comparison among different treatment groups of *Hyla arborea* tadpoles (W—white and B—black groups on the 20th day; WW—white–white, WB—white–black, BW—black–white and BB—black–black groups on the 37th day) for oxidative stress parameters

Variable	dF	F	p
*20th day*			
SOD	1	0.01	0.9369
CAT	1	**11.56**	**0.0032**
GSH-Px	1	3.35	0.0838
GR	1	3.33	0.0848
GST	1	1.61	0.2202
GSH	1	0.02	0.8820
LPO	1	2.08	0.1681
*37th day*			
SOD	3	**5.07**	**0.0042**
CAT	3	**3.68**	**0.0189**
GSH-Px	3	**14.45**	**< 0.0001**
GR	3	**16.26**	**< 0.0001**
GST	3	**5.22**	**0.0036**
GSH	3	**7.26**	**0.0005**
LPO	3	**14.16**	**< 0.0001**

Data in bold indicate statistical differences (p < 0.05)

**Fig. 4:**
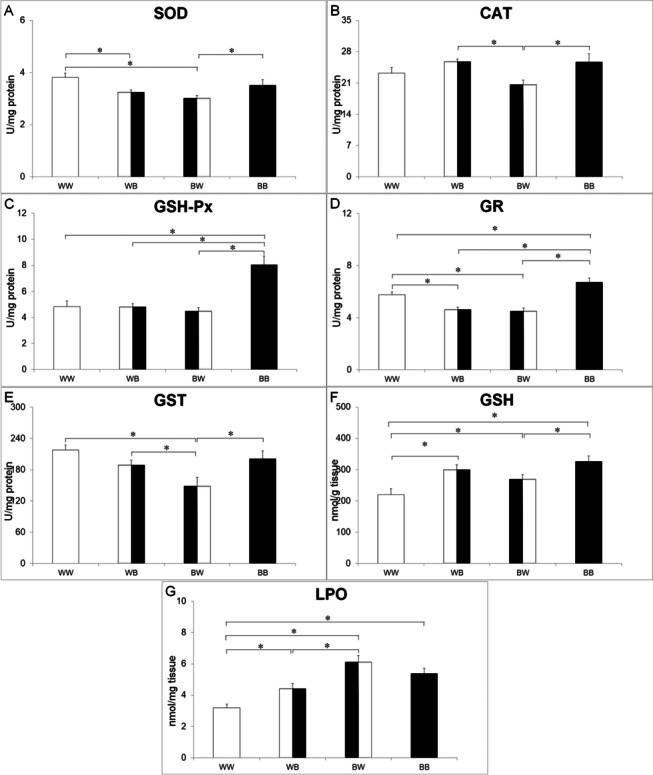
Antioxidant system (**A**—superoxide dismutase (SOD);** B**—catalase (CAT); **C**—glutathione peroxidase (GSH-Px); **D**—glutathione reductase (GR); **E**—glutathione-S-transferase (GST); **F**—glutathione (GSH)) and oxidative stress (**G**—lipid peroxidation (LPO)) parameters in four experimental groups (WW—white–white, WB—white–black, BW—black–white and BB—black–black) of *Hyla arborea* tadpoles after the background switch. *indicates significant differences between groups (Fisher LSD, p < 0.05)

Figure 4 presents the results of Fisher LSD post hoc test for oxidative stress parameters after prolonged background adaptation. SOD activity was significantly increased in the WW group compared to the WB (p = 0.0115) and BW groups (p = 0.0006), and also in the BB group as compared to group BW (p = 0.0271). In the BW group, CAT activity was significantly reduced compared to WB (p = 0.0067) and BB groups (p = 0.0072). In individuals that were continuously exposed to the black background (BB), the activity of GSH-Px was statistically increased in comparison to the other studied groups (BB vs. WW–p < 0.0001; BB vs. WB–p < 0.0001; BB vs. BW–p < 0.0001). The activity of GR was increased in the group of animals constantly subjected to the black background compared to the other groups (BB vs. WW–p = 0.0107; BB vs. WB–p < 0.0001; BB vs. BW–p < 0.0001), as it was in the group of animals constantly exposed to the white surroundings compared with groups WB (p = 0.0036) and BW (p = 0.015). The activity of GST was considerably lower in the BW group with respect to other investigated groups (BW vs. WW–p = 0.0004; BW vs. WB–p = 0.0329; BW vs. BB–p = 0.0064). The concentration of GSH in the WW group was lower than in all other groups (WW vs. WB–p = 0.0017; WW vs. BW–p = 0.0462; WW vs. BB–p < 0.0001), but it was enhanced in the BB group with respect to the BW group (p = 0.0218). The concentration of TBARS was the lowest in the WW group in comparison with other groups (WW vs. WB–p = 0.0135; WW vs. BW–p < 0.0001; WW vs. BB–p < 0.0001), and lower in the WB group in comparison to individuals from the BW group (p = 0.0008). TBARS values displayed a significant positive correlation with the intensity of coloration in individuals continuously maintained on dark and white backgrounds (r = 0.57; p = 0.016).

**Fig. 5:**
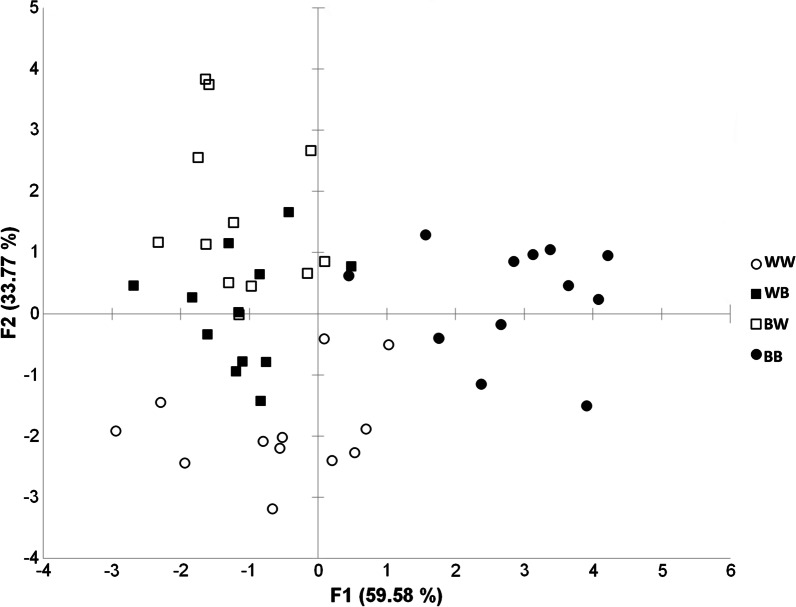
Discriminant analysis of investigated oxidative stress parameters in four experimental groups (WW—white–white, WB—white–black, BW —black–white and BB—black–black) of *Hyla arborea* tadpoles on the factor plan

Based on the measurements of oxidative stress components, discriminant analysis revealed differences between groups that developed on a background with different colors (WW, WB, BW and BB) after 37 days (Table 3; Fig. 5). The first function (F1) accounted for 59.58% of the total heterogeneity. The BB group was separated along the first function in relation to other groups, and was mostly differentiated regarding to GSH-Px and GR activities. The second function (F2) in the analysis accounted for 33.77% of the total heterogeneity. LPO concentration and SOD activity were the main factors that contributed to the differentiation of the WW group along the second canonical function in relation to the BW group.

**Table 3:** Standardized discriminant analysis coefficient for oxidative stress parameters in four experimental groups (WW—white–white, WB—white–black, BW—black–white and BB—black–black) of *Hyla arborea* tadpoles

	F1	F2	F3
SOD	0.2018	**− 0.5924**	− 0.1530
CAT	0.2683	− 0.1742	0.7147
GST	0.2208	− 0.5889	0.1794
GSH-Px	**0.8097**	0.0332	0.1356
GR	**0.7603**	− 0.3595	− 0.2165
GSH	0.3991	0.4062	0.6577
LPO	0.1646	**0.8561**	− 0.2060
Eigenvalue	2.9802	1.6892	0.3330
Discrimination (%)	59.5752	33.7674	6.6574
Cumulative %	59.5752	93.3426	100.0000

## Discussion

In many ectotherms, body color is susceptible to change and is fine-tuned at the individual level. Color adaptations require the formation, degradation, translocation and maintenance of pigments in chromatophores. These processes are energetically expensive and have significant implications on numerous physiological and biochemical processes. They can affect cortisol and corticosterone level [38, 39], body weight [40], morphology [14], standard metabolic rate and behavior [15]. Even though color change provides a direct benefit for animals in heterogeneous environments, it can allocate energy from other internal functions (antioxidant protection, the immune system) with effects on overall fitness [41]. It was found that background color affects the growth and survival of *Hippocampus kuda* juveniles [42] and the body length of *Microhylafissipes* tadpoles, suggesting that pigmentation is prioritized over somatic development [5].

In the present study, we assessed the effect of background color and its influence on body coloration and oxidative status parameters of anuran tadpoles. Our investigation revealed that tadpoles of *H. arborea* manifest the ability to adjust their body pigmentation to the background. A high-reflecting environment induced lower pigmentation, whereas a black, low-reflecting environment induced increased pigmentation.

Our assumption was that higher investment in pigmentation will result in an oxidative cost manifested as changes in the oxidative status and greater lipid peroxidation. Rearing larvae for 20 days on a dark (B) and light (W) substrate induced differences in body coloration, but did not affect antioxidant components (except CAT activity). The absence of significant differences in the concentration of LPO products indicates that investment in body pigmentation during this period did not cause greater oxidative stress in darker tadpoles of *H. arborea.* Larvae (early stages of development, GS 30) were capable of neutralizing potential variations in ROS formation at different substrate colors and successfully maintain homeostasis.

Nevertheless, prolonged exposure of larvae to a constantly dark habitat (for 37 days) led to major changes in the antioxidant status of individuals in the form of increased AOS activity and concentration of investigated parameters (GSH-Px, GR, GSH) as compared to the group kept continuously on a white background. Darker-pigmented larvae also exhibited greater oxidative damage (LPO). The level of oxidative damage showed a positive correlation with the color intensity of individuals. These results in *H. arborea* tadpoles could be the physiological consequences of the increase in the number/density of pigment cells and maintenance of a darker coloration for a prolonged time. Animals required to establish and maintain cryptic behavior in dark surroundings could experience elevated metabolic expenditure as a consequence of chromatic adaptation compared to animals in light surroundings. Previous studies suggested that individuals with higher pigmentations require greater food intake and encounter higher metabolic costs. [15] measured the standard metabolic rate (SMR) in newt larvae (*Lissotriton boscai*) subjected to different background conditions. After 14 days, larvae reared in a dark microhabitat exhibited a 64% increase in oxygen consumption in comparison with ones cultivated in a lighter substrate. A significant positive correlation between body color and SMR was obtained, and it was concluded that darker larvae displayed a higher percentage of oxygen consumption. In *Microhyla fissipes* tadpoles, a darker background caused oxidative phosphorylation and an overturn of protein metabolism, which was identified at the transcriptional level [5]. [43] assessed the oxidative status of *Lates calcarifer* juveniles in different colored tanks and observed that background colors did not induce significant variations in malondialdehyde, CAT or GST activities; however, SOD and GSH-Px activities were significantly elevated together with plasma cortisol in fish cultivated in black tanks, confirming stressful conditions.

Our investigation discloses that skin coloration of *H. arborea* tadpoles is reversible as individuals respond to changes in substrate hue. Exposure to altered substrate color can activate neural-hormonal events that require energy for pigment translocations within chromatophores. A dark background can enhance the transcription of proopiomelanocortin (POMC) and the expression of α-melanocyte-stimulating hormone (α-MSH), which controls the production of melanin and dispersion of melanosome in darker individuals [44]. These hormones also have an important role in the control of energy homeostasis [45]. The transfer to non-body-matching surroundings can cause pressure due to greater visibility and predation risk. This state activates CORT levels and the HPI-axis, which can also alter the energy budget and oxidative status.

In this study, we report on major changes in antioxidant protection parameters followed by variations in skin pigmentation. In individuals that reverse body coloration (BW group), the defense system (SOD, GR, GST) failed and LPO occurred in comparison to individuals reared continuously in the white surroundings. We observed consequences related to background color matching. The presence of oxidative damage in tadpoles that developed on altered substrates and in ones that were continuously maintained on a dark background suggests that the change in body coloration carries an oxidative cost similar to the one imposed by maintaining prolonged dark pigmentation.

Inverted body coloration as a response to a non-matching background can induce oxidative stress. However, color change from dark to lighter carries a higher oxidative cost. These individuals experienced both processes of melanin synthesis (aggregation) and destruction (dispersion), in contrast to only pigment production (aggregation) during the change from a pale to a darker coloration. Our results revealed that both processes involved in color change include oxidative stress as an associated cost.

It has been shown that pigments involved in appearance modification are also significant for other body functions related to the immunologic response [7, 12] or antioxidant protection [46]. A significant increase in pigment melanin in amphibian cells was noted in different stressful conditions such as oxygen and food deprivation or low temperature [47–50]. According to some data, melanin has a significant part in the elimination of ROS and other toxicants in amphibians and other species [50, 51]. Some authors even assume that the antioxidant role of SOD and GSH could be replaced with this pigment in amphibians [52, 53].

Even though body color modification is an effective tactic in many animals, its physiological repercussions have received limited attention. We deduce that alteration of background color induces very complex biological responses. These results indicate that amphibians encounter stress during chromatic adaptations, which eventually affects the components of their antioxidative defense.

## Conclusion

The influence of surrounding color tones on the behavior and physiology of animals is a field of science that is expanding and information concerning the effect of color on the development, fitness and survival of amphibians is of special concern. Examination of the ability of the amphibian population to cope with environmental variations, such as changes in substrate color, could help us understand how amphibians deal with background diversity and predict their susceptibility and resistance to these changes.

## Data Availability

The dataset used and analysed during the current study are available from the corresponding author on request.
